# The Effect of Iron Deficiency Anemia on Hemoglobin Glycation in Diabetics and Non-diabetics

**DOI:** 10.7759/cureus.70330

**Published:** 2024-09-27

**Authors:** Maddipatla Hari Krishna, Vijaykumar G Warad, Rahul Biradar

**Affiliations:** 1 General Medicine, Shri B. M. Patil Medical College Hospital and Research Centre, Bijapur Lingayat District Education (BLDE) (Deemed to be University), Vijayapura, IND

**Keywords:** anemia management, diabetes, glycemic control, hba1c, hemoglobin levels, iron deficiency anemia

## Abstract

Background

Iron deficiency anemia (IDA) is the most prevalent form of nutritional anemia worldwide, affecting a substantial portion of the population. It has significant negative effects on health and the economy, especially in poorer nations, and is more prevalent than anemia alone. Although hemoglobin A1c (HbA1c) is frequently used to track long-term glycemic management, several variables, including iron deficiency, might affect its values. For proper diabetic care, it is essential to comprehend this link. The purpose of this study is to evaluate the impact of IDA on HbA1c levels in individuals with and without diabetes.

Materials and methods

A hospital-based cross-sectional study was conducted at Shri B. M. Patil Medical College, BLDE (Deemed to be University). Group 1 consists of diabetic patients with IDA, Group 2 consists of diabetic patients without IDA, and Group 3 consists of non-diabetics with IDA. A total of 108 patients were involved in the study. A complete hemogram, anemia profile, and HbA1c values were also a part of the data-gathering process. IBM SPSS Statistics for Windows, Version 23 (Released 2015; IBM Corp., Armonk, New York) was used for the statistical analysis, with a significance level of p < 0.05.

Results

The study found no significant changes in the mean age, gender distribution, hypertension prevalence, weight, height, pulse rate, or blood pressure across the groups. Group 2 exhibited considerably higher hemoglobin levels than Groups 1 and 3. Group 1 showed substantially higher mean blood sugar and HbA1c levels than Group 3 (p < 0.05). Diabetic individuals had higher HbA1c levels than non-diabetic individuals, with the greatest levels found in diabetic patients with IDA.

Conclusion

This study found that IDA had a significant influence on HbA1c levels in diabetic patients, indicating that iron deficiency should be addressed when interpreting HbA1c levels in these individuals. These findings underscore the need for more study to better understand the processes behind this connection and the consequences for clinical management.

## Introduction

Iron deficiency anemia (IDA) is the most prevalent type of nutritional anemia worldwide. The World Health Organization (WHO) estimates that 2.1 billion individuals worldwide, or approximately 30% of the total population, are afflicted with IDA. Anemia is common in poor nations, with 39% in children under the age of five, 48% in children aged 5-14, 42% in women aged 15 to 59, 30% in males aged 15 to 59, and 45% in seniors over 60. These figures emphasize the serious economic and health consequences of anemia, particularly in low- and middle-income nations [1,2].

Anemia and iron deficiency cause severe productivity reductions. Iron deficiency in pregnant women is associated with preterm labor, low birth weight, and increased maternal and newborn death rates. It inhibits motor and cognitive development in youngsters, making them more susceptible to sickness. Anemia is a serious public health concern in India, affecting 70% of children aged 6-59 months, 55% of girls aged 15-49, and 24% of males aged 15-49, according to the National Family Health Survey (NFHS) [3,4]. Glycated hemoglobin, also known as hemoglobin A1c (HbA1c), is widely regarded as the gold standard for evaluating long-term glycemic control, reflecting blood sugar over the previous three months. The American Diabetes Association (ADA) recommends that diabetic people keep their HbA1c levels below 7% to avoid problems. HbA1c is also a useful early detection tool for common retinopathy.

The ADA and an International Expert Committee recommend utilizing the HbA1c test to diagnose diabetes. The WHO also recommends using HbA1c to diagnose diabetes as long as the tests are consistent and calibrated. A 2019 International Expert Committee advised diagnosing diabetes if HbA1c readings exceeded 6.5%, with further testing to confirm. Individuals with HbA1c values between 6.0% and 6.5% should take preventative actions since they are at a higher risk [5,6].

Early studies have shown a connection between IDA and HbA1c levels, with some indicating higher HbA1c levels in IDA patients, which decrease after treatment [7-9]. However, research findings have been inconsistent, and few studies have focused on the Indian population. This study aimed to evaluate HbA1c levels in IDA patients and observe changes in HbA1c levels following the correction of IDA. The objectives of the study are to measure the level of HbA1c among diabetic and non-diabetic patients, to identify the presence of iron deficiency among diabetic and non-diabetic patients, and to determine the effect of IDA on the HbA1c levels and compare diabetic and non-diabetic patients.

## Materials and methods

A cross-sectional descriptive study was carried out over a period of 18 months at the Bijapur Lingayat District Education (BLDE) (Deemed to be University) Shri B. M. Patil Medical College Hospital and Research Centre in Vijayapura. The study was categorized into three groups, comprising a total of 108 inpatients: Group 1 consists of diabetic patients with IDA, Group 2 includes diabetic patients without IDA, and Group 3 consists of non-diabetic patients with IDA.

Using the t-distribution with a 95% confidence level and 0.3 accuracy, the sample size was determined using JMP SAS 16 software (SAS Institute Inc., Cary, NC), assuming a 1.57 estimated population standard deviation. The sample size is 108 patients, which are divided into three groups, with each group comprising 36 patients.

The inclusion criteria required anemia patients with hemoglobin levels below 13 g/dL in men and 12 g/dL in women and serum ferritin levels below 9 ng/mL for women and 15 ng/mL for men. The exclusion criteria included glucose tolerance abnormalities, chronic alcohol ingestion, chronic renal failure, acute or chronic blood loss, hemolytic anemia, and vitamin B12 or folate deficiency.

Patients were selected based on these criteria, and their histories, clinical examinations, and investigations were documented. Ethical approval was obtained from the Institutional Ethics Committee (IEC-BLDE(DU)/IEC/750/2022-23). Complete blood counts were monitored upon admission, and anemic patients were further evaluated for serum ferritin and HbA1c levels. Blood parameters measured included a complete hemogram, hemoglobin (Hb), total count (TC), differential counts (DC), red blood cells (RBC), platelets, packed cell volume (PCV), anemia profile, peripheral smear, serum iron, transferrin iron-binding capacity (TIBC), serum ferritin, transferrin saturation, vitamin B12, folic acid levels, HbA1c, and urine analysis for albumin, sugar, and deposits.

The data were input into an Excel spreadsheet (Microsoft Corporation, Redmond, Washington) and analyzed with IBM SPSS Statistics for Windows, Version 23 (Released 2015; IBM Corp., Armonk, New York) on Windows 10. Tables, figures, bar graphs, and pie charts were used to depict summarized data in the form of mean, standard deviation, frequency, and percentages. ANOVA was used to evaluate mean differences in continuous data, while the chi-square test was used for categorical data. A p-value <0.05 was considered statistically significant.

## Results

In the present study, 108 patients meeting the inclusion criteria were divided into three groups. There is no significant difference in the mean age or gender distribution among the groups, although a general female preponderance is observed. The prevalence of hypertension is similar across all groups, and physical characteristics such as height and weight showed no significant differences. Vital parameters, including mean pulse rate and blood pressure, were also comparable between the groups (Tables 1, 2).

**Table 1:** Comparison of the mean parameters between the groups *The p-value is statistically significant MCV: mean corpuscular volume; TIBC: total iron-binding capacity; PR: pulse rate; SBP: systolic blood pressure; DBP: diastolic blood pressure; Hb: hemoglobin; RBS: random blood sugar; HbA1C: glycosylated hemoglobin; IDA: iron deficiency anemia

Parameters	Group 1 (Diabetic With IDA)		Group 2 (Diabetic Without IDA)		Group 3 (Non-diabetic With IDA)		p-value
	Mean	SD	Mean	SD	Mean	SD	
Age	53.72	7.3	54.9	6.8	55.01	6.1	0.651
MCV	65.93	8.37	65.54	8.4	68.09	7.32	0.51
Sr iron	20.54	5.53	73.35	8.71	18.54	5.62	0.01*
Ferritin	19.581	6.313	215.471	76.92	28.28	13.83	0.01*
TIBC	357.62	89.88	146.58	22.54	354.31	91.8	0.01*
PR	73.51	4.4	73.62	4.3	72.71	4.8	0.65
SBP	125.41	9.1	126.62	9.4	126.92	9.5	0.69
DBP	79.61	6.8	79.22	7.2	79.45	7.5	0.99
Hb	8.81	0.8	13.42	0.6	9.01	0.9	0.01*
RBS	275.34	25.52	224.01	28.9	165.87	7.13	0.01*
HbA1c	9.21	0.9	7.52	1	5.84	0.2	0.01*

**Table 2:** Comparison of gender and hypertension distribution between the groups %: percentage of the specified participants in the study group; N: number of participants in the study group; HTN: hypertension

Parameters		Group 1 (Diabetic With IDA)		Group 2 (Diabetic Without IDA)		Group 3 (Non-diabetic With IDA)	
		N	%	N	%	N	%
Gender	Female	21	58.30%	19	52.80%	20	55.60%
	Male	15	41.70%	17	47.20%	16	44.40%
HTN	Absent	17	47.20%	17	47.20%	16	44.40%
	Present	19	52.80%	19	52.80%	20	55.60%

However, significant differences are noted in the laboratory parameters: Groups 1 and 3 exhibited lower iron and ferritin levels compared to Group 2 and higher total iron-binding capacity (TIBC) compared to Group 2 (p < 0.05). Additionally, Group 2 exhibited significantly higher hemoglobin levels than Groups 1 and 3. Group 1 had a statistically significant higher mean blood sugar level than Group 3 (p < 0.05). When comparing HbA1c levels, Group 1 had significantly higher mean HbA1c levels than Groups 2 and 3.

## Discussion

HbA1c is hemoglobin with a glucose molecule attached to the β-chain's N-terminal valine. Normal adult hemoglobin includes HbF (0.5%), HbA2 (2.5%), and HbA (97%), with approximately 6% of HbA referred to as HbA1. HbA1 includes HbA1a1, HbA1a2, HbA1b, and HbA1c, with HbA1c being the most common. In healthy individuals, HbA1c makes up approximately 5% of total HbA [10]. While the physiological role of HbA1c is unclear, its levels are influenced by glucose concentration and protein half-life. Some clinical conditions, including IDA, have been associated with elevated HbA1c levels, and treatment of IDA has been shown to reduce these levels [11].

As per the data available, it has been suggested that IDA may be caused by a change in the quaternary structure of the hemoglobin molecule. This modification "will lead to an increase in the β-globin chain's glycosylation when the body is iron-deficient." IDA patients may have greater HbA1c levels due to the longer lifespan of RBC in circulation during the anemic state. Following iron therapy, bone marrow synthesis of RBC will rise, and new, immature RBC will be released, lowering HbA1c levels. There was a presupposition that there is a balance between hemoglobin concentration and HbA1c levels in individuals. Therefore, if the serum glucose remained constant, a decrease in hemoglobin concentration could cause an increase in the glycated fraction.

The interplay between IDA and HbA1c levels is crucial for diabetes management and diagnosis. HbA1c, which indicates long-term glucose control, can be influenced by IDA, a common nutritional anemia impacting many, particularly in low- and middle-income countries. IDA may affect HbA1c levels by altering erythrocyte lifespan and glycation rates, making accurate diabetes assessment challenging.

The present study of 108 patients revealed significantly higher HbA1c levels in those with diabetes and IDA compared to those without IDA and also in non-diabetic individuals with IDA. These findings align with several studies that have documented elevated HbA1c levels in IDA patients. For instance, Brooks et al. noted increased HbA1c in IDA individuals, which decreased with iron therapy, suggesting that iron deficiency may enhance hemoglobin glycosylation [12]. Similarly, Christy et al. found elevated HbA1c in iron-deficient individuals and emphasized the need to consider IDA before adjusting diabetes treatment [11]. Contrarily, Hong et al. found that IDA only shifted HbA1c levels in normoglycemic and prediabetic ranges, not into diabetic ranges, indicating that IDA should be considered in HbA1c screening for prediabetes and diabetes [13].

Comparatively, Silva et al. found that the severity of anemia affects HbA1c levels, suggesting that mild anemia has a minor impact on HbA1c1 [14]. Koga et al. and other studies observed varying associations between anemia indices and HbA1c, emphasizing the complex relationship between IDA and HbA1c levels [15]. The current study's results highlight the importance of integrating iron status assessments into diabetes management to improve the accuracy of HbA1c measurements and ensure more informed treatment decisions.

Limitations

The relatively small sample size of 108 patients may limit the generalizability of the findings to broader populations, as it might not adequately represent diverse demographics. Additionally, the cross-sectional design restricts the ability to establish causal relationships between IDA and HbA1c levels, as data were collected at a single point in time. The study was conducted in a specific hospital setting, which could introduce selection bias and may not reflect the experiences of patients in different geographic or socioeconomic contexts. Furthermore, there are potential confounding factors, such as comorbidities, medications, and lifestyle variations. Lastly, variability in laboratory techniques for measuring HbA1c and iron parameters might affect the consistency and accuracy of results. These limitations suggest that further research with a larger, more diverse cohort and a longitudinal design is necessary to deepen the understanding of how IDA influences HbA1c levels in both diabetic and non-diabetic populations.

## Conclusions

These results highlight the significant impact of diabetes and IDA on HbA1c levels and iron profile parameters, indicating a crucial interaction between these IDA and HbA1c. HbA1c is notably higher among diabetic patients compared to non-diabetic patients, with the highest in diabetic patients with IDA. Further research is needed to better understand these relationships and their implications for patient management and treatment strategies.
